# Effects of anodal tDCS on resting state eeg power and motor function in acute stroke: a randomized controlled trial

**DOI:** 10.1186/s12984-023-01300-x

**Published:** 2024-01-03

**Authors:** O. Vimolratana, B. Aneksan, V. Siripornpanich, V. Hiengkaew, T. Prathum, W. Jeungprasopsuk, T. Khaokhiew, R. Vachalathiti, W. Klomjai

**Affiliations:** 1 Faculty of Physical Therapy, Mahidol University, 999 Phuttamonthon 4 Road, Salaya, Nakhon Pathom, 73170 Thailand; 2 Neuro Electrical Stimulation Laboratory, Faculty of Physical Therapy, Mahidol University, Nakhon Pathom, 73170 Thailand; 3 School of Integrative Medicine, Mae Fah Luang University, Chiang Rai, 57100 Thailand; 4 Institute of Molecular Biosciences, Mahidol University, Nakhon Pathom, 73170 Thailand; 5 Faculty of Medical Technology, Mahidol University, Nakhon Pathom, 73170 Thailand

**Keywords:** Acute, Stroke, Cardiovascular disease, Transcranial direct current stimulation, Physical therapy, Motor training, Rehabilitation, EEG

## Abstract

**Background:**

Anodal transcranial direct current stimulation (tDCS) is a beneficial adjunctive tool in stroke rehabilitation. However, only a few studies have investigated its effects on acute stroke and recruited only individuals with mild motor deficits. This study investigated the effect of five consecutive sessions of anodal tDCS and conventional physical therapy on brain activity and motor outcomes in individuals with acute stroke, with low and high motor impairments.

**Methods:**

Thirty participants were recruited and randomly allocated to either the anodal or sham tDCS group. Five consecutive sessions of tDCS (1.5 mA anodal or sham tDCS for 20 min) were administered, followed by conventional physical therapy. Electroencephalography (EEG), Fugl-Meyer Motor Assessment (FMA), and Wolf Motor Function Test (WMFT) were performed at pre-, post-intervention (day 5), and 1-month follow-up. Sub-analyses were performed on participants with low and high motor impairments. The relationship between EEG power and changes in motor functions was assessed.

**Results:**

Linear regression showed a significant positive correlation between beta bands and the FMA score in the anodal group. Elevated high frequency bands (alpha and beta) were observed at post-intervention and follow-up in all areas of both hemispheres in the anodal group, while only in the posterior area of the non-lesioned hemisphere in the sham group; however, such elevation induced by tDCS was not greater than sham. Lower limb function assessed by FMA was improved in the anodal group compared with the sham group at post-intervention and follow-up only in those with low motor impairment. For the upper limb outcomes, no difference between groups was found.

**Conclusions:**

Five consecutive days of anodal tDCS and physical therapy in acute stroke did not result in a superior improvement of beta bands that commonly related to stroke recovery over sham, but improved lower extremity functions with a post-effect at 1-month follow-up in low motor impairment participants. The increase of beta bands in the lesioned brain in the anodal group was associated with improvement in lower limb function.

*Trial registration*: NCT04578080, date of first registration 10/01/2020.

**Supplementary Information:**

The online version contains supplementary material available at 10.1186/s12984-023-01300-x.

## Background

Neuronal cell death after stroke leads to fluctuations in neural oscillation in both the lesioned and non-lesioned hemispheres recorded by electroencephalography (EEG) [1, 2]. High-frequency EEG powers (alpha and beta bands) were reduced after stroke [2]. A reduction in functional connectivity in the lesioned hemisphere is associated with poor functioning, which can indicate stroke severity [3, 4]. For example, beta oscillations were diminished after stroke both at rest and during movement and this was more apparent in stroke individuals with high motor impairment [5]. Increased beta-band activity in the motor area of the lesioned hemisphere during the early period after stroke onset (2–3 weeks) was observed in those with better motor function in the sub-acute phase [6]. Moreover, improved motor outcomes are also associated with an increase in alpha-band functional connectivity in the lesioned hemisphere [2, 7]. As this regard, high-frequency EEG powers (alpha and beta bands) appears as a predictive tool for motor recovery post-stroke.

The early period after stroke onset is crucial for enhancing recovery in individuals with stroke, especially within the first month [8]. Early rehabilitation has been recommended to enhance recovery in individuals with stroke, particularly within the first 2 weeks [9, 10]. However, some motor deficits may remain even after an intensive rehabilitation program. Additional treatments i.e., non-invasive brain stimulation techniques (NIBS) have been recommended to facilitate post-stroke motor recovery as it can modulate cortical excitability with positive long-lasting effect [11]. Most commons NIBS that have been used in individuals with stroke are transcranial direct current stimulation (tDCS) and repetitive transcranial magnetic stimulation (rTMS). Both techniques have shown similar moderate effects in stroke rehabilitation [12]; however, a recent meta-analysis showed that tDCS is superior to rTMS in improving gait, balance, and lower limb function in stroke populations [13]. Moreover, tDCS is portable and practical to use at the bedside, which allows to use in an acute stroke unit setting. To promote motor recovery, tDCS is often applied over the primary motor area (M1). Within dose limits, tDCS can modulate cortical excitability with polarity-dependent: anodal tDCS enhances cortical excitability, whereas cathodal tDCS decreases it [14–16]. Moreover, anodal tDCS over the M1 has been reported to enhance high-frequency EEG powers. For example, a single session of anodal tDCS (1.75 mA, 20 min with 35-cm^2^ electrical pad) increases beta and alpha bands in chronic stroke individuals [17]. Similar observations have been reported after a single session of anodal tDCS (1 mA, 20 min with 35-cm^2^ electrical pad) in healthy subjects [18]. Regarding performance levels, anodal tDCS combined with motor training increased upper and lower extremity functions in individuals with subacute and chronic stroke [19–21]. tDCS effects have been reported in various phases of stroke. However, several meta-analyses have reported limited evidence regarding the application of tDCS in the acute phase of stroke [22–24]. Moreover, most tDCS studies in acute stroke recruited individuals with mild motor deficits, with no reporting of neurophysiological assessments (i.e., cortical activity) [25–27]. Stroke people with lower motor impairment may response better to tDCS than those with higher impairment [28, 29], possibly due to residual M1 cortical excitability [30, 31]. Therefore, tDCS study in acute stroke with varied levels of impairment may help to develop more efficient therapy strategies to overcome stroke deficits.

A meta-analysis from studies using at least five sessions of tDCS has suggested that a higher charge or current density or smaller electrode size is associated with greater efficacy in post-stroke upper extremity motor recovery [32]. As commonly used tDCS electrodes are sized between 25 and 35 cm^2^ [33], the smallest one was selected. Here, we investigated the effects of five consecutive sessions of anodal tDCS (1.5 mA, for 20 min with a 25-cm^2^ electrical pad) combined with conventional physical therapy. We assessed cortical activity and clinical measures of upper and lower limb functions in acute stroke participants with high and low motor impairments at before (baseline), after the 5-day intervention, and at 1-month follow-up. We hypothesized that compared with conventional physical therapy alone, five consecutive daily sessions of anodal tDCS combined with conventional physical therapy would higher increase high-frequency EEG power (i.e., alpha and beta bands) and motor functions in individuals with acute stroke.

## Methods

### Participants

Thirty-four individuals with acute stroke were recruited from the acute stroke unit of Siriraj Hospital, Bangkok, Thailand from January 2021 to May 2022. The inclusion criteria were as follows: age between 18 and 75 years with first acute ischemic stroke of the anterior circulation system within 2–10 days, stable medical condition, conscious and alert, able to follow commands, and a Modified Rankin Scale (mRS) score of ≤ 4. Participants were excluded if they had a hemorrhagic stroke, recurrent stroke, unilateral neglect, pusher syndrome, other neurological disorders (e.g., normal pressure hydrocephalus), contraindication to tDCS (i.e., metal implantation, intracranial shunt, cardiac pacemakers, open or infectious wound around the scalp, history of epilepsy), or any factor that could interfere with EEG (i.e., ischemic heart disease, peripheral vascular ischemia, late-stage kidney or liver disease, body mass index > 30 kg/m^2^, or undergoing hormonal treatment [34–37]). All medications were recorded; none of the participants received any medications that could affect tDCS efficacy (i.e., sodium and calcium channel blockers) [38]. Self-reported handedness, by asking participants which hand they prefer to use to perform a task, was used to determine dominant handedness and recorded in the demographic data.

### Experimental protocol

**Fig. 1:**
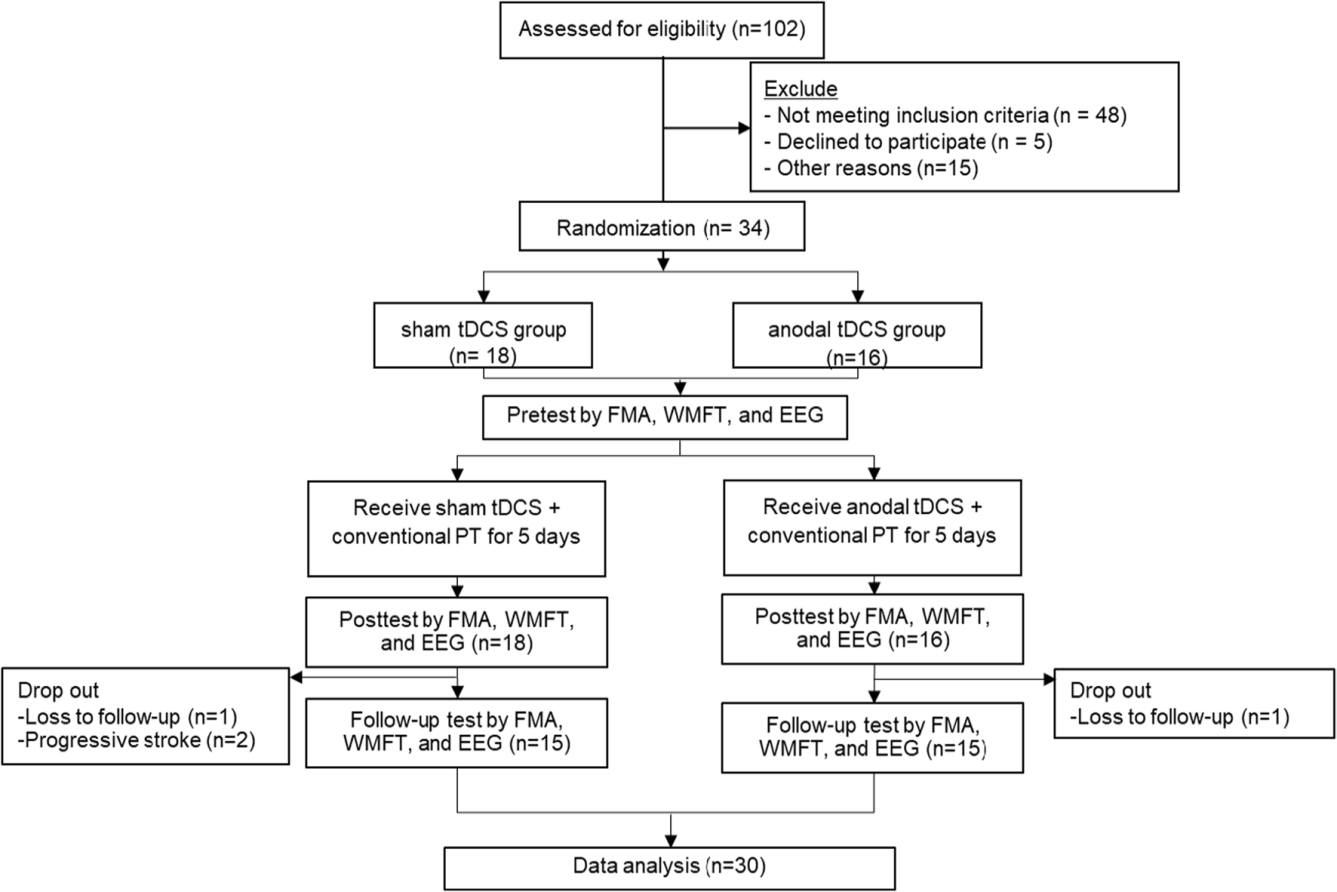
Flowchart of the study

This study was a double-blinded randomized control trial. Participants who met the inclusion criteria were randomly allocated into two groups, namely anodal or sham tDCS groups, using concealed envelopes. Randomization was managed by an independent researcher not involved in the intervention and evaluation of the outcomes. Participants were matched for at least two out of three criteria (stroke severity assessed using the National Institute of Health Stroke Scale score (NIHSS), lesion, and age) in each pair. Sham blinding was also performed by an independent researcher by programming tDCS parameter to either active or sham mode. Participants, assessors, and physical therapists were unaware of the group allocation and blinding process. All participants were evaluated before the intervention (PRE), after the intervention on day 5 (POST), and at 1-month follow-up (F/U) using EEG, Fugl-Meyer Assessment (motor domain) (FMA), and Wolf Motor Function Test (WMFT). The resting state EEG was evaluated, followed by FMA and WMFT. All participants were evaluated with the same order of assessment. The intervention lasted 5 days for all participants. On day 1, all participants underwent pre-testing followed by resting for at least 15 min or until they recovered from fatigue. Subsequently, tDCS was administered during the resting state, while sitting for 20 min, followed by a conventional physical therapy program based on participants’ impairments for 30–40 min. On days 2–4, the intervention (i.e., tDCS for 20 min followed by a conventional physical therapy program for 30–40 min) was administered. On day 5, the same intervention was administered, followed by a post-test assessment. Participants were instructed to rest between treatment and assessment for at least 15 min or until they recovered from fatigue. All participants were scheduled for a follow-up assessment 1 month after the intervention. Adverse effects of tDCS were monitored during and after each session of intervention. The flowchart of the study is illustrated in Fig. 1.

The present study was approved by the Mahidol University Central Institutional Review Board (MU-CIRB 2018/238.0712) and registered on ClinicalTrials.gov (ID NCT04578080, date of first registration 10/01/2020). The study was conducted following The Code of Ethics of the World Medical Association (Declaration of Helsinki) for experiments involving humans. The study procedure and group allocation were explained to all participants before participating and written informed consent for study participation and publication of the results were provided from all participants.

### Intervention

#### tDCS

Anodal tDCS (Ybrain, MINDD STIM; Seongnam-si, Gyeonggi-do, Republic of Korea) was administered at 1.5 mA for 20 min (with ramp up and down for 30 s each) before the rehabilitation program via two rectangular saline-soaked sponge pads (25 cm^2^). The international 10–20 EEG system was used to locate the M1 position of the ipsilesional hemisphere. The anode was placed over C3/C4 of the lesioned hemisphere, while the cathode was placed over the supraorbital area of the contralateral hemisphere (Fp1/Fp2). For the sham tDCS group, the sham mode was set as ramp up and down for 30 s each. Electrical stimulation was automatically turned off after ramping up while the electrodes remained in position with an active beeping sound for 20 min.

#### Rehabilitation program

A conventional physical therapy program based on participants’ impairment was provided immediately following tDCS for 30–40 min. For those with high motor impairment, standing balance training and gait training were not performed. The program was as follows: (1) stretching exercise of both upper limb (elbow flexor, wrist flexor, and shoulder flexor) and lower limb muscles (hip extensor, knee flexor, and ankle plantar flexor); (2) strength exercise of both upper limb (shoulder flexor, shoulder abduction, elbow extensor, wrist extensor, and finger extensor) and lower limb muscle (hip extensor, hip abductor, knee flexor, and ankle dorsiflexor); (3) bed-mobility training; (4) unimanual upper limb functional training: reach to grasp exercise; (5) balance training; (6) gait training.

### Motor outcome measurements

#### FMA (motor domain)

Prior to evaluation, participants were allowed to practice using their unaffected side to avoid the learning effects. Each task was repeated three times, and the best trial was selected. The best performance was scored through direct observation as follows: 0 = could not perform, 1 = performed partially, and 2 = performed fully.

The total score was 100 (66 and 34 points for the upper and lower limbs, respectively). FMA motor domain is used to evaluate upper and lower extremity functions in supine, sitting, and standing positions [39] and is a suitable predictor of motor recovery in acute stroke [40].

#### WMFT

To avoid fatigue, the present study assessed only 2 tasks (lifting a can and lifting a pencil) from WMFT. Given several muscles and joints are involved, visual guidance is required, and these tasks are commonly used in daily life, their sensitivity to change is not unusual. Improving in the lift can and lift pencil tasks are feasible and challenging enough to represent greater changes of overall WMFT score in stroke population [41]. All tasks were performed as quickly as possible and truncated at 120 s [42]. WMFT has acceptable reliability, validity, and responsiveness to change over time in the acute stroke population [43]. Participants were in a sitting position when instructed to perform each task, which was demonstrated by an assessor beforehand. Each participant was allowed to practice a few times on their unaffected extremities before the first trial and repeat the task 3 times. An assessor recorded the best time to complete the task in seconds.

#### Quantitative electroencephalogram (qEEG)

Resting-state EEG is a reliable biomarker that may help with screening in stroke [44–46]. Closed-eye resting-state EEG can examine spontaneous brain activity unbiased by any task. A Waveguard^™^ 32-electrode EEG cap (ANT Neuro, Hengelo, The Netherlands) and eego sport^™^ software were used. To maintain the position of the EEG electrodes (Fp1, Fp2, Fpz, Fz, F3, F4, F7, F8, Fc1, Fc2, Fc5, Fc6, Cz, C3, C4, T7, T8, CP1, CP2, CP5, CP6, Pz, P3, P4, P7, P8, POz, Oz, O1, and O2) over the scalp during different measurements, the length from the nasion to inion and right to left preauricular points was noted in all participants. During data collection, participants were instructed to relax, refrain from speaking or moving, and avoid any cognitive or mental tasks while keeping their eyes closed for 5 min.

The scalp was cleaned, and CZ was identified before EEG cap placement. Participants were instructed to avoid using gel or hair spray on the testing day to decrease impedance during measurements. Conductive electrode gel was inserted into each electrode. Impedance was checked and maintained below 5 kΩ throughout data collection. The average of both mastoid areas (M1 + M2)/2 was used as the recording reference. The low pass filter was set at 0.03 Hz, while the high pass filter at 60 Hz. The notch filter was set at 50 Hz to reduce powerline artifacts. Raw EEG data were recorded with a sampling rate of 1,000 Hz. The analog–digital converter was set at 500 Hz.

EEG data were preprocessed offline using ANT 4.10.1. A Butterworth bandpass with filter steepness at 24 dB/oct (decibels per octave) was used. The low-band pass filter of 30 Hz and a high-band pass filter of 0.5 Hz were applied to all EEG data. The notch filter was set at 50 Hz. Automatic artefacts detection was set at ± 100 μV amplitudes. Artefacts were removed with automatic preprocessing in all EEG epochs, and manually preprocessed. The continuous EEG file was cut into 2-s-interval EEG epochs followed by Fast Fourier transformation (FFT) by asa^™^ (ANT Neuro, Netherlands) to acquire the absolute power of all frequency bands as follows: delta (1–4 Hz), theta (4.1–8 Hz), alpha (8.1–12.5 Hz), and beta (12.6–30 Hz).

### Statistical analysis

The demographic characteristics (Table 1) and tDCS-related side effects reported by participants were analyzed using descriptive statistics. For EEG analysis, raw absolute power (μV^**2**^) of each frequency band (delta, theta, alpha, and beta) was averaged across respective electrodes of the region of interest (ROI)*:* frontal (left hemisphere: FP1, F3, and F7; right hemisphere: FP2, F4, and F8), central (left hemisphere: C3, Cp5, Cp1, FC1, and FC5; right hemisphere: C4, CP6, CP2, FC2, and FC6), and posterior (left hemisphere: P3 and O1; right hemisphere: P4 and O2). As there were lesions in both hemispheres, data were categorized into the lesioned and non-lesioned hemispheres. Therefore, the averaged raw absolute power (μV^**2**^) from each ROI of each hemisphere was used for statistical analysis.

**Table 1:** Characteristics of all participants

Characteristics	Anodal tDCS (n = 15)	Sham tDCS (n = 15)	*p*-value
Age (years)^α^	52.53 (15.05)	62.27 (9.68)	0.046^a^
Gender (female/male)^β^	9/6	7/8	0.714^c^
Handedness (right/left)^β^	13/2	11/4	0.651^d^
NIHSS scores	5 (4.00;8.00)	6 (2.00;8.00)	0.629^b^
Onset since stroke (days)	4.00 (3.00;6.00)	4.00 (4.00;9.00)	0.510^b^
Lesion area (Cortical to subcortical/subcortical)^β^	4/11	3/12	1.000^d^
tDCS stimulation side (right brain/left brain)^β^	6/9	10/5	0.272^d^
Stroke severity by FMA-UE^β^			
-No UE capacity (0–22)	6	5	–
-Poor capacity (23–31)	1	1	–
-Limited capacity (32–47)	–	2	–
-Notable capacity (48–52)	–	1	–
-Full UE capacity (> 53)	8	6	–

Statistical analysis by using

^a^Independent t-test

^b^Mann-Whitney U test

^c^Chi-square test

^d^Fisher’s exact test

^α^Data are presented in Mean (SD)

Data are presented in median (Q1;Q3)

^β^Data are presented in number

For statistical analysis of motor outcomes [FMA and WMFT], absolute change scores (∆) from individual PRE data were used for analysis, and the calculated formulas were as follows: (1) baseline =|PRE-PRE|, (2) at POST =|POST–PRE|, and 3) at F/U =|F/U-PRE|. For sub-analysis, the pretest FMA-UE scores were used to categorize participants into two groups, namely low and high motor impairments, as all participants had middle cerebral artery infarction, which usually affected UE > LE [47]. Based on the upper extremity categories [48], participants with FMA-UE scores < 53 were categorized into the high motor impairment group, and those with FMA-UE scores ≥ 53 were categorized into the low motor impairment group. Sub-analysis was performed to compare the anodal and sham groups in participants with low and high motor impairments.

The normality of the data was first tested using the Shapiro–Wilk test. Between-group (anodal vs. sham) and within-group (pre vs. post vs. F/U) comparisons were performed using mixed analysis of variance (ANOVA) if the data were normally distributed. For non-normally distributed data, between-group comparison was performed using the Mann–Whitney *U* test, and within-group comparison was performed using the Friedman test. Statistical significance was set at *p* < 0.05. Multiple post-hoc comparisons using the Bonferroni correction were performed if any significant main effect or interaction effect was observed. Bonferroni’s correction [*p* 0.05/3 = 0.016] was applied for multiple comparison. In addition, both the between-and within group effect size were calculated from the mean and SD using Cohen’s formula [49, 50]. Cohen’s *d*-values of 0.20, 0.50, and 0.80 were interpreted as small, moderate, and large effect sizes, respectively [49].

To clarify the effect of demographic characteristics (i.e., age and sex) on the outcomes, a two-way mixed analysis of covariance (ANCOVA) was used for between-group comparison. In addition, a linear regression model was used to test the association between the change in spectral power and motor outcomes (FMA and WMFT).

## Results

**Fig. 2:**
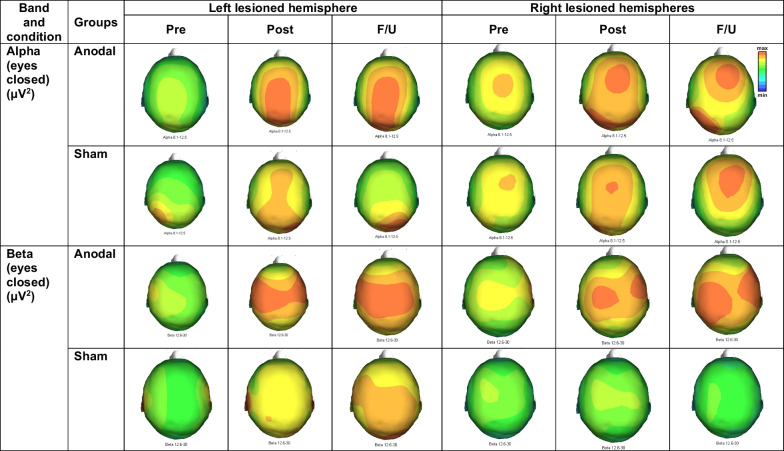
Average topographical map of alpha and beta band during eyes closed condition in left and right lesioned hemispheres

**Fig. 3:**
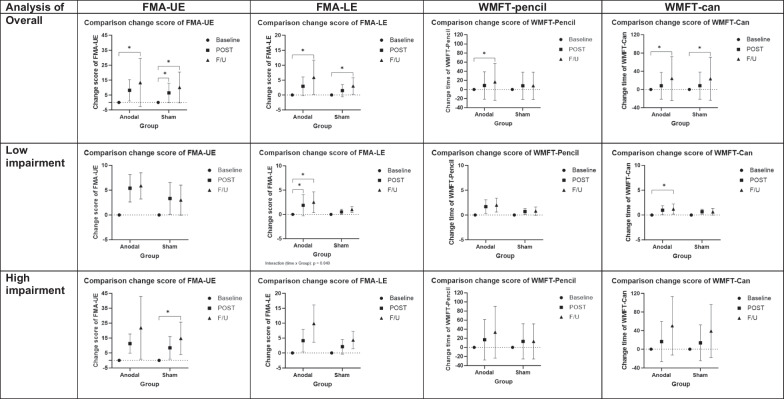
Comparisons of delta change of motor outcomes in anodal and sham groups

Participant characteristics are presented in Table 1. The topographical map of qEEG data is illustrated in Fig. 2. All the qEEG data is presented in Table 2 and Additional file 1: Datas S1–S4. The FMA and WMFT results are presented in Fig. 3 and Additional file 1: Data S5.

**Table 2:** Absolute power of alpha and beta in the frontal, the central, and the posterior area during eyes closed

Areas	Hemispheres	Bands	Groups	Means (SD)/median (Q1; Q3)			Within group comparison *p*-value (effect size)				Between groups comparison *p*-value (effect size)				Interaction effect (Time x Group)
				Pre	Post	F/U	Overall	Pre vs Post	Pre vs F/U	Post vs F/U	Overall	At pre	At Post	At F/U	
Frontal	Lesioned	Alpha	Anodal	7.97 (4.69;25.67)	13.60 (3.95;33.07)	11.53 (7.19;28.79)	0.005	0.053 (0.37)	0.006* (0.37)	1.000 (0.04)	–	0.158 (0.06)	0.455 (0.28)	0.245 (0.44)	–
			Sham	14.16 (10.14;18.05)	16.53 (11.88;29.20)	19.26 (13.67;30.82)	0.127	–	–	–					
		Beta	Anodal	6.67 (4.22;11.81)	6.68 (5.41;13.51)	9.11 (6.66;12.48)	0.155	–	–	–	–	0.455 (0.40)	0.803 (0.10)	0.125 (0.59)	–
			Sham	6.31 (5.39;9.36)	7.92 (5.57;9.62)	7.71 (4.75;9.32)	0.936	–	–	–					
	Non-lesioned	Alpha	Anodal	7.30 (4.59;24.53)	12.68 (3.95;30.07)	9.05 (7.11;28.02)	0.002	0.010* (0.38)	0.006* (0.37)	1.000 (0.02)	–	0.158 (0.13)	0.300 (0.39)	0.184 (0.51)	–
			Sham	12.94 (10.25;17.14)	20.91 (12.46;26.86)	20.43 (13.64;31.27)	0.155	–	–	–					
		Beta	Anodal	7.08 (4.18;9.26)	7.96 (4.90;15.37)	8.54 (6.83;10.84)	0.008	0.204 (0.51)	0.006* (0.64)	0.604 (0.09)	–	0.934 (0.09)	0.967 (0.02)	0.431 (0.30)	–
			Sham	7.11 (4.85;7.84)	7.52 (5.55;10.77)	7.55 (6.05;9.58)	0.344	–	–	–					
Central	Lesioned	Alpha	Anodal	14.11 (5.39;28.43)	22.07 (4.06;34.35)	13.05 (10.66;39.23)	0.041	0.085 (0.44)	0.085 (0.36)	1.000 (0.08)	–	0.320 (0.03)	0.836 (0.08)	0.407 (0.31)	–
			Sham	15.46 (11.35;22.49)	22.29 (12.68;27.84)	21.85 (15.65;31.09)	0.189	–	–	–					
		Beta	Anodal	7.75 (5.94;10.55)	9.00 (6.78;14.93)	11.26 (8.28;14.81)	0.038	0.604 (0.29)	0.032 (0.60)	0.604 (0.35)	–	0.590 (0.44)	0.740 (0.13)	0.074 (0.70)	–
			Sham	7.046 (3.77;10.84)	10.04 (5.60;13.12)	9.28 (4.81;12.41)	0.344	–	–	–					
	Non-lesioned	Alpha	Anodal	10.46 (6.04;25.71)	19.59 (4.26;38.04)	13.63 (10.52;34.90)	0.002	0.010* (0.44)	0.006* (0.38)	1.000 (0.05)	–	0.263 (0.18)	0.431 (0.30)	0.199 (0.49)	–
			Sham	17.11 (11.03;27.42)	26.31 (16.13;28.76)	28.95 (17.00;34.98)	0.038	0.134 (0.62)	0.053 (0.70)	1.000 (0.02)					
		Beta	Anodal	7.57 (4.73;9.15)	8.61 (8.96;14.55)	9.49 (7.26;14.36)	< 0.001	0.019 (0.48)	< 0.001* (0.66)	0.820 (0.20)	–	0.934 (0.04)	0.967 (0.02)	0.455 (0.28)	–
			Sham	8.33 (4.09;11.02)	9.03 (6.00;13.32)	9.98 (6.02;12.24)	0.420	–	–	–					
Posterior	Lesioned	Alpha	Anodal	14.90 (7.97;23.28)	22.18 (4.12;45.81)	15.68 (9.19;53.40)	0.038	0.053 (0.53)	0.134 (0.45)	1.000 (0.05)	–	0.934 (0.22)	0.709 (0.14)	0.740 (0.13)	–
			Sham	13.83 (9.40;22.18)	17.80 (10.43;35.68)	18.78 (14.52;34.86)	0.085	–	–	–					
		Beta	Anodal	5.61 (4.54;9.55)	7.01 (6.09;9.29)	7.99 (6.94;11.54)	0.002	0.134 (0.36)	0.002* (0.57)	0.432 (0.19)	–	0.619 (0.35)	0.967 (0.02)	0.561 (0.22)	–
			Sham	5.99 (3.21;9.11)	7.82 (4.47;10.57)	8.00 (4.25;10.93)	0.127	–	–	–					
	Non-lesioned	Alpha	Anodal	14.72 (7.64;19.76)	18.92 (5.35;53.24)	15.59 (8.41;42.43)	0.002	0.003* (0.55)	0.019 (0.45)	1.000 (0.09)	–	0.868 (0.04)	0.740 (0.13)	0.361 (0.35)	–
			Sham	13.85 (9.00;28.32)	22.61 (15.36;41.57)	27.66 (14.11;44.21)	0.005	0.053 (0.70)	0.006* (0.77)	1.000 (0.15)					
		Beta	Anodal	5.71 (4.41;11.18)	7.75 (5.94;9.51)	8.51 (7.10;10.67)	< 0.001	0.019 (0.38)	< 0.001* (0.58)	0.820 (0.23)	–	0.868 (0.24)	1.000 (0.01)	0.836 (0.08)	–
			Sham	6.75 (3.23;8.73)	7.11 (4.40;13.58)	7.77 (5.69;13.52)	0.031	0.204 (0.68)	0.032 (0.75)	1.000 (0.15)					

^*^Statistically significant after Bonferroni’s correction (*p* < 0.016), Bolded number indicate statistically significant, Cohen’s values of 0.20, 0.50, and 0.80 were interpreted as small, moderate, and large effect sizes, respectively

There was no significant difference between the two groups in the baseline characteristics except for age (Table 1). For the adverse effect, mild adverse effects were observed, including a tingling sensation (anodal 73.33% vs. sham 6.67%, *p* < 0.001), itching (anodal 66.67% vs. sham 0%, *p* < 0.001), redness beneath the electrode (anodal 13.33% vs. sham 0%, *p* > 0.05), headache (anodal 13.33% vs. sham 0%, *p* > 0.05), and burning sensation (anodal 6.67% vs. sham 0%, *p* > 0.05).

### qEEG

#### High-frequency bands

- Alpha band

As shown in Table 2, a comparison between group showed no significant difference between anodal vs. sham groups (*p* > 0.05) at POST and F/U. There was no significant difference in the alpha band at baseline. For within-group comparison, Friedman’s test with Bonferroni correction revealed significant enhancements of alpha bands within the anodal group in all brain regions of both hemispheres, while it was increased only in the posterior of the non-lesioned hemisphere in the sham group. Post-hoc comparisons (PRE vs. POST vs. F/U) with p-value correction, and effect size data were reported in Table 2.

No significant differences between-group were observed for sub-analysis (*p* > 0.05).

- Beta band

As shown in Table 2, a comparison between group showed no significant difference between anodal vs. sham groups (*p* > 0.05) at POST and F/U. There was no significant difference in the beta band at baseline. For within-group comparison, Friedman’s test with Bonferroni correction revealed significant enhancements of beta bands within the anodal group in all brain regions of the non-lesioned hemisphere and in the posterior area of the lesioned hemisphere. No significant difference was found in the sham group after Bonferroni correction. Post-hoc comparisons (PRE vs. POST vs. F/U) with p-value correction, and effect size data were reported in Table 2.

No significant differences between-group were observed for sub-analysis (*p* > 0.05).

#### Low-frequency bands

No significant differences were observed in both groups for delta and theta bands (*p* > 0.05).

#### Motor outcome measurements

- FMA-UE

No significant differences between groups for overall analysis and sub-analysis (*p* > 0.05). Within-group analysis showed improvements for both groups. Post-hoc comparisons with p-value correction, and effect size data were reported in Additional file 1: Data S5.

- FMA-LE

No significant difference between groups was found for overall analysis (*p* > 0.05). Within-group analysis showed improvements for both groups. Post-hoc comparisons with p-value correction, and effect size data were reported in Additional file 1: Data S5.

For sub-analysis, two-way mixed ANOVA revealed a significant interaction between time and groups effect (F (2, 24) = 3.690, *p* = 0.040). Post-hoc comparison with Bonferroni correction showed a significant improvement at post (*p* = 0.005, with large effect size (Cohen’s *d* = 0.81)) and F/U (*p* < 0.001) only in the anodal groups in participants with low motor impairment. There was no significant difference between groups in participants with high motor impairment (*p* > 0.05).

- WMFT-pencil

No significant difference within- and between-groups were found for overall analysis and sub-analysis (*p* > 0.05).

- WMFT-can

There were improvements in both groups, however no significant difference between groups was found for overall analysis and sub-analysis (*p* > 0.05).

### Association between EEG response and motor outcomes

**Fig. 4:**
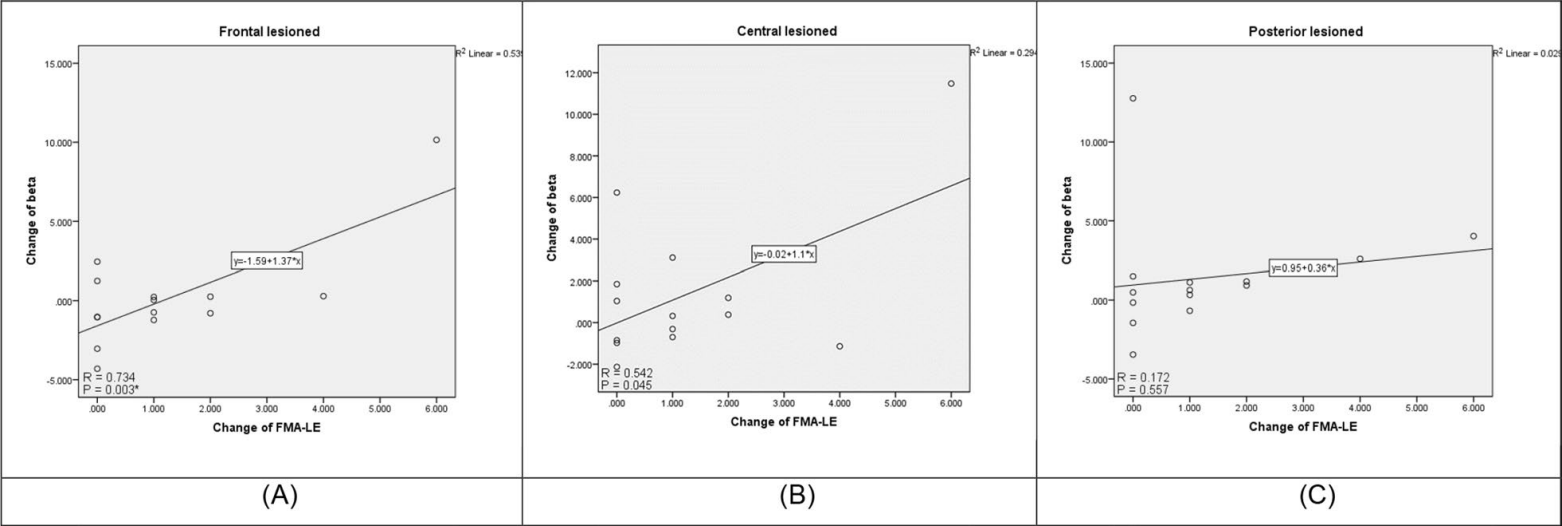
Relation between EEG response and motor outcomes. Scatter plots of ∆ change (|post–pre|) of beta bands and FMA-LE at the frontal area (**A**), the central area (**B**), and the posterior area (**C**) of the lesioned brain in low motor impairment participants in the anodal group

An increased beta band induced by anodal tDCS was associated with FMA-LE score in participants with low impairment as shown by the linear regression model with Bonferroni corrected p-values. The relation was found in the frontal area of the lesioned hemisphere (*p* < 0.003) (Fig. 4). No significant relation was found in the non-lesion hemisphere (*p* > 0.05).

### Impact of demographic characteristics on outcomes

Two-way mixed ANCOVA demonstrated that age and sex did not influence the motor outcomes and EEG data (*p* > 0.05). For subgroup analysis, there was no effect of age and sex on motor outcomes and all absolute frequency bands.

(*p* > 0.05) in both low and high motor impairment groups. There was no significant difference between the anodal and sham tDCS groups in the proportion of left and right hemispheric lesions (*p* > 0.05).

## Discussion

We investigated the effects of anodal tDCS combined with conventional rehabilitation for 5 consecutive days on motor functions and brain activity in individuals with acute stroke. EEG data did not support the hypothesis of this study since no significant difference was found between anodal vs. sham groups. However, elevated high frequency bands (alpha and beta) were observed in both hemispheres (frontal, central, and posterior) in the anodal group at post-intervention and follow-up, while it was only observed in the non-lesioned hemisphere (central and posterior) in sham group. Only data from the lower limb motor performance supported the hypothesis. In participant with low impairment, the anodal group showed a greater improvement of the lower limb function evaluated by FMA-LE over the sham group at post-intervention and follow-up with large effect sizes.

Besides that, the increase of beta bands of the lesioned brain in the anodal group showed an association with FMA-LE. For the upper limb motor outcomes evaluated by FMA-UE and WMFT, no difference changes between groups were observed.

### EEG(μV^2^)

We expected an increase in the high frequency bands following anodal tDCS combined with motor training. Our findings showed an increase in alpha and beta bands in both lesioned and non-lesioned hemispheres, but such improvement was not over sham. Enhancement of high-frequency bands (alpha and beta) over the frontal and central have been reported after motor training during post-stroke recovery phases, which are related to motor relearning and recovery process [7, 44, 51–53], reflecting a better motor recovery [44, 54]. Enhancement of high-frequency bands (alpha and beta) have also been reported to increase following anodal tDCS. For example, in healthy participants, Mangia et al. have reported that a single session of 1.5 mA anodal tDCS for 15 min over the postero-parietal cortex (P4) enhances alpha and beta absolute power beneath the stimulated site and remote from the stimulated site, including the non-stimulated brain [55]. Moreover, they found that EEG power was much more sensitive to tDCS stimulation in the eye-closed condition than in the eye-opened condition, that was probably due to a higher processing capability to tDCS available during eye-closed as in resting state the signal power is higher in the eye-closed condition [56]. This is agreed with our study in term of widespread activation of alpha and beta bands in several brain regions during eye-closed following tDCS, although the stimulation site was difference. In individuals with chronic stroke, Wang et al. have shown that a session of 1.75-mA anodal tDCS over the lesioned M1 for 20 min enhances alpha frequency bands in the frontal, central and parietal region of the lesioned hemisphere during eye-opened condition [17], but no reports in the eye-closed condition. Moreover, they also reported that 1.75-mA bilateral-tDCS over both M1 cortices for 20 min could enhance alpha and beta bands, while changes in beta bands had a positive correlation with the FMA score. This is in line with our study that found a positive relation between beta bands in the lesioned hemisphere and FMA score. Although, it could not directly compare since we explored EEG in different eye conditions, but in the eye-opened condition that the EEG power may be less sensitive, their results in the chronic stroke showed the same trend as our in the acute phase.

The mechanism underlying the modulation of high-frequency bands is controversial [57]. However, a possible mechanism may involve the interaction between cellular GABAergic-glutamatergic neurons in the cortex [44]. GABA concentration influences alpha and beta bands [58, 59]. Compared with healthy individuals, reduced GABA levels in M1 have been reported during acute [60] and chronic stroke recovery [61]. GABA levels in affected M1 hemispheres can be increased by motor training, which was associated with motor improvement in individuals with acute ischemic stroke [60]. Moreover, tDCS modulates the level of glutamatergic [62, 63] and GABAergic neurons in the cortex [64]. Das et al. have proposed that the anodal tDCS effect elevates glutamate and GABA concentrations in the cortex by sub-threshold depolarization [65]. Both motor training and tDCS have positive effects on neurobiological changes post-stroke.

Herein, no significant change in the low-frequency band (i.e., delta power) was observed. Delta is associated with the deafferentation of neuronal activity. Giaquinto et al. have reported a significant reduction in delta absolute power at 3- and 6-month follow-ups in individuals with stroke [52]. This may explain the unchanged delta absolute power observed in the present study within 1-month post-stroke.

### Motor outcomes

The anodal group showed a positive effect compared with the sham group on the lower limb function (evaluated using FMA-LE) in low motor impairment participants with after-effects for at least 1-month post-intervention. No benefit of anodal tDCS over sham tDCS was observed in the upper limb evaluated by FMA-UE and WMFT. It was reported that the rate of recovery of the lower limb was greater than that of the upper limb especially during the first 4-week post-stroke [66], and also the more severe impairment, the longer period of recovery [67]. This could possibly explain a limited effect on motor function found in the present study. However, it should be noted that the anodal stimulation site was C3/C4, which is more related to the upper-limb M1, but the observed motor improvement was found for the lower-limb. Previous studies have observed that tDCS applied over the C3/C4 influences both upper- and lower-limb performance [25, 26, 68]. This may be caused by the non-focality of tDCS [69, 70].

Regarding the effect of tDCS with motor training in individuals with acute stroke, the improvement in motor outcomes observed in the present study is consistent with the results of previous studies. In acute stroke, Sattler et al. have reported better improvement in upper extremity function after five consecutive sessions of anodal tDCS (1.2 mA for 13 min with 35 cm^2^-electrical pad, total charge density at 0.04 mAh/cm^2^) combined with repetitive peripheral nerve stimulation (rPNS), and the post-effect was maintained for 1 month [71]. Bornheim et al. have shown better motor recovery following 20 sessions of physical therapy with anodal tDCS (2 mA for 20 min with 25-cm^2^ electrical pad, total charge density at 0.53 mAh/cm^2^) over physical therapy alone, and its effects lasted for 1 year [25]. We have previously reported the benefit of five consecutive sessions of physical therapy with anodal tDCS (1.5 mA for 20 min with 35-cm^2^ electrical pad, total charge density at 0.07 mAh/cm^2^) on lower extremity function in low motor impairment participants with acute stroke, and its effect lasted for 1 month [26]. It could suggest that anodal tDCS for at least 5 sessions appears to have positive effects on motor performance in acute stroke.

### High vs. low motor impairment

A greater improvement of lower extremity in the anodal group was limited only to low motor impairment participants. This is consistent with the motor outcome results obtained in the meta-analysis of Marquez et al. that reported a better motor response to tDCS in individuals with stroke with mild to moderate motor severity [72]. Lin et al. have shown a higher inhibition from the non-lesioned hemisphere toward the lesioned hemisphere in individuals with stroke with high motor impairment (FMA-UE < 43), while there was less inhibition from the non-lesioned hemisphere in those with low motor impairment (FMA-UE ≥ 43) [73]. Moreover, a greater residual M1 cortical excitability were reported in those with low motor impairment [30, 31].Therefore, participants with low motor impairment may have more preserved neurons in the lesioned hemispheres to respond to anodal stimulation.

### tDCS application in post-stroke phases

Different stages of stroke recovery cause differences in response to tDCS. Pavlova et al. have explored a direct comparison between subacute and chronic stroke stages following tDCS combined with upper extremity training and observed a better enhancement of upper extremity functions in individuals in the subacute than the chronic phases [19]. A meta-analysis has reported beneficial long-term learning effects after tDCS with training in both subacute and chronic recovery stages post-stroke, with a slightly higher effect size in the subacute stage [28]. Nevertheless, tDCS also benefits chronic stroke in which the spontaneous recovery of the brain is reduced [74, 75]. Another meta-analysis has also reported the dominant benefit of tDCS on the recovery of upper extremity functions in chronic stroke [76]. To date, evidence regarding the effect of tDCS in acute stroke compared with other phases is scarce. Our results could not confirm whether providing tDCS earlier during the spontaneous recovery phase would promote better recovery in individuals with stroke compared with other phases.

### Limitations of the study

First, we recruited participants with acute stroke of various severity to generalize the findings; however, when the sub-analysis was performed, there were a small number of participants in each group (6–9 participants). Hence, a higher number of participants is recommended for future studies. Second, the baseline of theta bands was different between the two groups at the frontal and central of the lesioned and non-lesioned hemispheres (Additional file 1: Data S1). Stroke is associated with an increased low-frequency band (i.e., theta) [77] and its increased activity suggests an unfavorable recovery post-stroke [78]. Although our study found unchanged theta throughout the experiment, this point should be cautious in further studies. Third, there was no restriction on conventional rehabilitation during the follow-up period, and a logbook was provided for all participants to record the rehabilitation program. The type of training between the two groups did not differ (Additional file 1: Data S6). Fourth, the follow-up period was only 1 month; the time at which the after-effect ends remains to be determined. A longer follow-up period is recommended as the highest recovery rate is reportedly observed during the first 3 to 6 months post-stroke [79]. Fifth, age had a marginal statistically significant difference between the two groups (*p* < 0.046), although it was one of the factors used for match-pairing between groups (anodal 52.53(15.05) vs. sham 62.27(9.68) years old). Aging is related to anatomical changes and brain connectivity [80, 81]. However, in participants with stroke aged < 70 years, a significant improvement in motor recovery was observed within 6 months and could improve up to 30 months post stroke onset [82]. Age and sex had no influence on EEG and motor outcomes in the present study. Sixth, limitations associated with WFMT. Although the WFMT was a quantitative measure of the upper extremity, it allowed for only 120 s to perform a task. Most of the participants with high motor impairment could not complete the tasks within that time, but the value 120 s was obligated to be used in the analysis. Therefore, WMFT tasks remains incomplete in some participants. Lastly, we reported a high rate of tDCS-related sensations, such as tingling and itching, for the anodal group, which was not experienced by the sham group and we did not ensure the effectiveness of blinding. There is a probability of correctly identifying active or sham tDCS, especially with a high intensity (i.e., 2 mA or higher) [83, 84]. Whereas, for low intensity (1 mA), sham tDCS was not distinguishable from active tDCS regarding the perception of stimulation strength and assessment of stimulation type in both naive and experienced subjects [85]. However, a previous tDCS study using 1.5 mA (the same as in the present study) showed that participants’ beliefs of having received the active or sham tDCS had no impact on task performance [86].

## Conclusions

Anodal tDCS for five consecutive sessions combined with conventional physical therapy is beneficial for improving lower-limb performance in acute ischemic stroke individuals with low motor impairment, with a positive post-effect at 1-month post-intervention, but induced no changes in brain activity compared with sham. However, the observed improvement of the lower limb function was associated with an increase in beta bands in the lesioned hemisphere commonly related to motor learning and recovery.

### Supplementary Information

**Additional file 1: Data S1.** Absolute power of delta and theta in the frontal, the central, and the posterior area during eyes closed. **Data S2.** High frequency of absolute power sub-analysis (alpha and beta) in the frontal area. **Data S3.** High frequency of absolute power sub-analysis (alpha and beta) in the central area. **Data S4.** High frequency of absolute power sub-analysis (alpha and beta) in the posterior area. **Data S5.** Means raw scores of motor outcomes (FMA-UE, FMA-LE, WMFT-pencil, and WMFT-can) and statistical analysis. **Data S6.** Summary of received rehabilitation details after intervention were shown in Table A. Fisher exact test reported no significant difference between the groups.

## Data Availability

The datasets analyzed during the current study are available in the OSF platform (https://osf.io/5rmjc/).
