# Supplementary material for: Effects of anodal tDCS on resting state eeg power and motor function in acute stroke: a randomized controlled trial
Source: J Neuroeng Rehabil. 2024 Jan 3;21:6. doi: 10.1186/s12984-023-01300-x (PMC10765911; doi:10.1186/s12984-023-01300-x)
Supplement: Supplementary file 1 — Additional file 1: Data S1. Absolute power of delta and theta in the frontal, the central, and the posterior area during eyes closed. Data S2. High frequency of absolute power sub-analysis (alpha and beta) in the frontal area. Data S3. High frequency of absolute power sub-analysis (alpha and beta) in the central area. Data S4. High frequency of absolute power sub-analysis (alpha and beta) in the posterior area. Data S5. Means raw scores of motor outcomes (FMA-UE, FMA-LE, WMFT-pencil, and WMFT-can) and statistical analysis. Data S6. Summary of received rehabilitation details after intervention were shown in Table A. Fisher exact test reported no significant difference between the groups. [file 12984_2023_1300_MOESM1_ESM.docx]

**Additional data 1** Absolute power of delta and theta in the frontal, the central, and the posterior area during eyes closed.

| **Areas** | **Hemispheres** | **Bands** | **Groups** | **Means (SD)/median (Q1; Q3)** | | | **Within group comparison**  ***p*-value (effect size)** | | | | **Between groups comparison**  ***p*-value (effect size)** | | | | **Interaction effect (Time x Group)** |
| --- | --- | --- | --- | --- | --- | --- | --- | --- | --- | --- | --- | --- | --- | --- | --- |
|  |  |  |  | **Pre** | **Post** | **F/U** | **Overall** | **Pre vs Post** | **Pre vs F/U** | **Post vs F/U** | **Overall** | **At pre** | **At Post** | **At F/U** |  |
| Frontal | Lesioned | Delta | Anodal | 30.03  (20.20) | 34.50  (23.43) | 41.06  (20.88) | 0.929 | - | - | - | 0.567 | - | - | - | 0.011 |
|  |  |  | Sham | 46.54  (31.65) | 39.73  (29.87) | 32.63  (17.29) |  | - | - | - |  |  |  |  |  |
|  |  | Theta | Anodal | 11.17  (4.52;16.61) | 13.73  (4.90;24.07) | 13.67  (4.85;16.45) | 0.057 | - | - | - | - | 0.023  (0.78) | 0.619  (0.19) | 0.648  (0.18) | - |
|  |  |  | Sham | 19.93  (9.58;26.06) | 13.42  (9.35;25.56) | 12.37  (10.15;14.97) | 0.282 | - | - | - |  |  |  |  |  |
|  | Non-lesioned | Delta | Anodal | 27.10  (16.74) | 28.94  (16.11) | 35.22  (18.35) | 0.801 | - | - | - | 0.432 | - | - | - | 0.050 |
|  |  |  | Sham | 36.69  (22.33) | 39.43  (30.99) | 30.05  (12.07) |  | - | - | - |  |  |  |  |  |
|  |  | Theta | Anodal | 10.54  (4.44;14.15) | 11.54  (5.08;25.36) | 10.62  (4.353;16.11) | 0.085 | - | - | - | - | 0.023  (0.88) | 0.361  (0.35) | 0.245  (0.44) | - |
|  |  |  | Sham | 17.064  (9.52;25.80) | 11.447  (8.82;32.43) | 11.644  (10.66;17.99) | 0.549 | - | - | - |  |  |  |  |  |
| Central | Lesioned | Delta | Anodal | 14.91  (10.58;24.17) | 17.64  (13.12;30.79) | 19.17  (12.63;26.34) | 0.627 | - | - | - | - | 0.229  (0.59) | 0.740  (0.13) | 0.740  (0.13) | - |
|  |  |  | Sham | 29.68  (12.60;43.00) | 19.25  (13.48;26.51) | 21.41  (12.86;27.25) | 0.627 | - | - | - |  |  |  |  |  |
|  |  | Theta | Anodal | 10.48  (4.04;15.03) | 12.76  (5.22;17.09) | 11.88  (4.43;17.36) | 0.074 | - | - | - | - | 0.038  (0.72) | 0.384  (0.33) | 0.281  (0.41) | - |
|  |  |  | Sham | 20.79  (10.33;23.20) | 18.08  (8.08;21.70) | 12.31  (10.11;15.16) | 0.344 | - | - | - |  |  |  |  |  |
|  | Non-lesioned | Delta | Anodal | 14.51  (9.98;21.83) | 17.86  (14.03;28.79) | 21.17  (12.91;26.60) | 0.074 | - | - | - | - | 0.158  (0.49) | 0.934  (0.04) | 0.772  (0.11) | - |
|  |  |  | Sham | 24.59  (12.52;32.62) | 20.13  (12.98;27.45) | 18.67  (12.76;23.72) | 0.247 | - | - | - |  |  |  |  |  |
|  |  | Theta | Anodal | 9.72  (3.74;14.75) | 11.04  (5.42;17.53) | 10.82  (4.38;16.60) | **0.008** | **0.006***  (0.23) | 0.604  (0.04) | 0.204  (0.19) | - | 0.051  (0.70) | 0.384  (0.33) | 0.384  (0.33) | - |
|  |  |  | Sham | 18.76  (8.98;22.89) | 12.04  (7.45;27.66) | 11.39  (7.65;16.82) | 0.344 | - | - | - |  |  |  |  |  |
| Posterior | Lesioned | Delta | Anodal | 13.86  (10.27;22.55) | 13.70  (11.26;24.88) | 17.98  (10.09;19.68) | 0.627 | - | - | - | - | 0.213  (0.55) | 1.000  (0.01) | 0.967  (0.02) | - |
|  |  |  | Sham | 23.88  (8.84;37.63) | 15.89  (11.53;24.60) | 15.70  (10.13;25.12) | 0.074 | - | - | - |  |  |  |  |  |
|  |  | Theta | Anodal | 7.75  (3.63;14.39) | 9.12  (5.84;12.42) | 8.43  (4.17;13.21) | 0.247 | - | - | - | - | 0.097  (0.34) | 0.431  (0.30) | 0.431  (0.30) | - |
|  |  |  | Sham | 13.86  (6.64;17.27) | 14.26  (5.88;16.57) | 9.57  (6.99;18.40) | 0.936 | - | - | - |  |  |  |  |  |
|  | Non-lesioned | Delta | Anodal | 11.49  (8.54;19.56) | 14.12  (12.48;27.34) | 17.27  (10.57;22.63) | 0.155 | - | - | - | - | 0.199  (0.40) | 0.836  (0.08) | 0.836  (0.08) | - |
|  |  |  | Sham | 19.48  (9.22;24.61) | 16.89  (11.13;23.70) | 15.01  (11.13;25.10) | 0.344 | - | - | - |  |  |  |  |  |
|  |  | Theta | Anodal | 7.39  (3.39;14.35) | 7.99  (5.15;13.86) | 8.86  (4.18;11.27) | 0.057 | - | - | - | - | 0.106  (0.45) | 0.678  (0.16) | 0.561  (0.22) | - |
|  |  |  | Sham | 12.17  (6.43;17.01) | 9.30  (5.65;20.09) | 8.92  (6.51;17.32) | 0.420 | - | - | - |  |  |  |  |  |

*Statistically significant after Bonferroni’s correction (*p* < 0.016), Bolded number indicate statistically significant.

**Additional data 2** High frequency of absolute power sub-analysis (alpha and beta) in the frontal area.

| **Hemisphere** | **Bands** | **Analysis of** | **Groups** | **Means (SD)/median (Q1;Q3)** | | | **Within group comparisons**  ***p*-value (effect size)** | | | | **Between groups comparisons**  ***p*-value (effect size)** | | | | **Interaction effect**  **(Time x Group)** |
| --- | --- | --- | --- | --- | --- | --- | --- | --- | --- | --- | --- | --- | --- | --- | --- |
|  |  |  |  | **Pre** | **Post** | **F/U** | **Overall** | **Pre vs Post** | **Pre vs F/U** | **Post vs F/U** | **Overall** | **At pre** | **At post** | **At F/U** |  |
| **Lesioned** | **Alpha** | **Low** | **Anodal** | 11.91  (14.84) | 17.37  (18.28) | 16.71  (20.38) | 0.012 | - | - | - | 0.456 | - | - | - | 0.909 |
|  |  |  | **Sham** | 18.49  (7.38) | 22.61  (10.61) | 23.12  (10.27) |  | - | - | - |  |  |  |  |  |
|  |  | **High** | **Anodal** | 16.66  (11.55) | 22.96  (19.20) | 25.54  (23.37) | 0.013 | - | - | - | 0.543 | - | - | - | 0.999 |
|  |  |  | **Sham** | 12.31  (4.48) | 18.72  (12.38) | 21.48  (14.59) |  | - | - | - |  |  |  |  |  |
|  | **Beta** | **Low** | **Anodal** | 7.49  (3.63;11.96) | 8.29  (4.70;14.33) | 10.62  (5.10;12.92) | 0.236 | - | - | - | - | 1.000  (0.09) | 1.000  (0.00) | 0.414  (0.50) | - |
|  |  |  | **Sham** | 9.27  (5.54;10.87) | 8.29  (5.65;9.07) | 7.65  (5.64;9.57) | 0.570 | - | - | - |  |  |  |  |  |
|  |  | **High** | **Anodal** | 6.67  (5.23;11.35) | 6.68  (5.72;13.51) | 8.98  (7.46;10.84) | 0.768 | - | - | - | - | 0.244  (0.79) | 0.832  (0.35) | 0.204  (0.35) | - |
|  |  |  | **Sham** | 5.66  (4.95;8.25) | 7.62  (5.24;12.38) | 6.93  (4.22;14.44) | 0.569 | - | - | - |  |  |  |  |  |
| **Non-lesioned** | **Alpha** | **Low** | **Anodal** | 11.48  (14.25) | 16.84  (17.55) | 16.45  (20.41) | 0.005 | - | - | - | 0.370 | - | - | - | 0.993 |
|  |  |  | **Sham** | 18.54  (7.77) | 24.28  (11.23) | 23.73  (10.27) |  | - | - | - |  |  |  |  |  |
|  |  | **High** | **Anodal** | 15.83  (11.26) | 22.20  (19.21) | 23.49  (20.26) | 0.007 | - | - | - | 0.738 | - | - | - | 0.919 |
|  |  |  | **Sham** | 12.42  (5.28) | 20.07  (13.39) | 22.29  (14.35) |  | - | - | - |  |  |  |  |  |
|  | **Beta** | **Low** | **Anodal** | 6.42  (4.02;10.18) | 7.48  (4.45;16.57) | 8.71  (5.51;12.44) | 0.285 | - | - | - | - | 0.852  (0.17) | 0.950  (0.07) | 0.755  (0.21) | - |
|  |  |  | **Sham** | 7.46  (5.64;9.64) | 8.51  (5.88;11.55) | 7.88  (5.71;10.35) | 0.252 | - | - | - |  |  |  |  |  |
|  |  | **High** | **Anodal** | 7.08  (4.18;8.02) | 7.96  (4.90;11.23) | 8.54  (7.29;9.88) | **0.008** | 0.855  (0.41) | **0.010***  (0.60) | 0.184  (0.17) | - | 0.916  (0.29) | 0.916  (0.42) | 0.525  (0.07) | - |
|  |  |  | **Sham** | 6.96  (4.78;7.77) | 7.22  (5.21;11.92) | 7.55  (5.04;12.84) | 0.917 | - | - | - |  |  |  |  |  |

*Statistically significant after Bonferroni’s correction (*p* < 0.016), Bolded number indicate statistically significant.

**Additional data 3** High frequency of absolute power sub-analysis (alpha and beta) in the central area.

| **Hemisphere** | **Bands** | **Analy-sis of** | **Groups** | **Means (SD)/median (Q1;Q3)** | | | **Within group comparisons**  ***p*-value (effect size)** | | | | **Between groups comparisons**  ***p*-value (effect size)** | | | | **Inter-action effect**  **(Time x Group)** |
| --- | --- | --- | --- | --- | --- | --- | --- | --- | --- | --- | --- | --- | --- | --- | --- |
|  |  |  |  | **Pre** | **Post** | **F/U** | **Overall** | **Pre vs Post** | **Pre vs F/U** | **Post vs F/U** | **Overall** | **At pre** | **At post** | **At F/U** |  |
| **Lesioned** | **Alpha** | **Low** | **Anodal** | 14.63  (17.34) | 23.36  (22.12) | 21.06  (23.04) | 0.016 | - | - | - | 0.704 | - | - | - | 0.444 |
|  |  |  | **Sham** | 20.94  (7.27) | 24.45  (7.45) | 23.89  (9.09) |  | - | - | - |  |  |  |  |  |
|  |  | **High** | **Anodal** | 15.23  (12.73;28.43) | 22.07  (14.62;34.35) | 15.10  (11.83;43.74) | 0.305 | - | - | - | - | 0.672  (0.60) | 0.751  (0.19) | 0.832  (0.13) | - |
|  |  |  | **Sham** | 15.11  (10.83;19.24) | 17.61  (10.25;29.81) | 18.01  (12.41;37.63) | 0.328 | - | - | - |  |  |  |  |  |
|  | **Beta** | **Low** | **Anodal** | 8.87 (4.87;14.36) | 10.37  (6.66;15.77) | 13.12  (6.15;20.39) | 0.794 | - | - | - | - | 0.755  (0.15) | 0.662  (0.28) | 0.228  (0.74) | - |
|  |  |  | **Sham** | 10.53  (6.22;11.52) | 9.71  (6.45;12.21) | 9.16  (5.48;11.72) | 0.956 | - | - | - |  |  |  |  |  |
|  |  | **High** | **Anodal** | 9.08  (4.37) | 10.97  (7.78) | 13.75  (7.77) | 0.007 | - | - | - | 0.356 | - | - | - | 0.657 |
|  |  |  | **Sham** | 6.37  (3.17) | 9.67  (5.17) | 10.18  (6.78) |  | - | - | - |  |  |  |  |  |
| **Non-lesioned** | **Alpha** | **Low** | **Anodal** | 13.78  (15.95) | 21.43  (20.28) | 19.80  (23.29) | 0.007 | - | - | - | 0.467 | - | - | - | 0.860 |
|  |  |  | **Sham** | 21.34  (9.36) | 26.79  (9.16) | 26.20  (9.44) |  | - | - | - |  |  |  |  |  |
|  |  | **High** | **Anodal** | 13.01 (10.15;25.71) | 20.95  (12.45;38.04) | 16.00  (10.82;40.49) | 0.085 | - | - | - | - | 0.916  (0.31) | 0.916  (0.08) | 0.751  (0.19) | - |
|  |  |  | **Sham** | 16.24  (10.27;19.52) | 26.80  (10.63;28.36) | 17.81  (12.34;41.07) | 0.107 | - | - | - |  |  |  |  |  |
|  | **Beta** | **Low** | **Anodal** | 7.88  (4.46) | 11.43 (7.44) | 13.37 (8.85) | 0.041 | - | - | - | 0.639 | - | - | - | 0.097 |
|  |  |  | **Sham** | 9.17  (4.14) | 9.60 (3.73) | 9.63 (3.60) |  | - | - | - |  |  |  |  |  |
|  |  | **High** | **Anodal** | 8.02  (5.17;9.10) | 8.61  (5.96;10.44) | 9.40  (7.22;11.61) | **0.004** | 0.326  (0.33) | **0.004***  (0.47) | 0.326  (0.14) | - | 0.832  (0.30) | 1.000  (0.03) | 0.832  (0.13) | - |
|  |  |  | **Sham** | 7.40  (3.79;11.11) | 9.03  (4.34;14.41) | 9.73  (5.52;14.83) | 0.814 | - | - | - |  |  |  |  |  |

*Statistically significant after Bonferroni’s correction (*p* < 0.016), Bolded number indicate statistically significant.

**Additional data 4** High frequency of absolute power sub-analysis (alpha and beta) in the posterior area.

| **Hemisphere** | **Bands** | **Analysis of** | **Groups** | **Means (SD)/median (Q1;Q3)** | | | **Within group comparisons**  ***p*-value (effect size)** | | | | **Between groups comparisons**  ***p*-value (effect size)** | | | | **Interaction effect**  **(Time x Group)** |
| --- | --- | --- | --- | --- | --- | --- | --- | --- | --- | --- | --- | --- | --- | --- | --- |
|  |  |  |  | **Pre** | **Post** | **F/U** | **Overall** | **Pre vs Post** | **Pre vs F/U** | **Post vs F/U** | **Overall** | **At pre** | **At post** | **At F/U** |  |
| **Lesioned** | **Alpha** | **Low** | **Anodal** | 18.08  (20.82) | 31.18  (30.15) | 28.57  (30.81) | 0.049 | - | - | - | 0.790 | - | - | - | 0.740 |
|  |  |  | **Sham** | 18.24  (7.06) | 24.88  (16.91) | 25.33  (16.25) |  | - | - | - |  |  |  |  |  |
|  |  | **High** | **Anodal** | 17.21  (6.38) | 26.06  (17.80) | 26.20  (19.29) | 0.020 | - | - | - | 0.476 | - | - | - | 0.942 |
|  |  |  | **Sham** | 13.54  (7.42) | 22.38  (17.30) | 20.57  (10.58) |  | - | - | - |  |  |  |  |  |
|  | **Beta** | **Low** | **Anodal** | 6.65  (1.11;13.26) | 7.89  (4.81;14.03) | 9.17  (7.09;14.60) | **0.047** | 0.634  (0.35) | 0.037  (0.55) | 0.634  (0.18) | - | 0.950  (0.15) | 0.950  (0.07) | 0.662  (0.28) | - |
|  |  |  | **Sham** | 8.39  (4.78;9.43) | 7.91  (4.64;9.84) | 7.40  (5.60;13.71) | 0.570 | - | - | - |  |  |  |  |  |
|  |  | **High** | **Anodal** | 5.61  (4.96;7.12) | 6.57  (6.34;8.59) | 7.36  (5.59;11.54) | 0.051 | - | - | - | - | 0.397  (0.50) | 1.000  (0.50) | 0.832  (0.35) | - |
|  |  |  | **Sham** | 5.00  (3.17;6.89) | 7.48  (4.42;13.59) | 8.00  (4.07;12.46) | 0.278 | - | - | - |  |  |  |  |  |
| **Non-lesioned** | **Alpha** | **Low** | **Anodal** | 17.73  (18.74) | 27.78  (26.28) | 21.81  (21.86) | 0.029 | - | - | - | 0.724 | - | - | - | 0.512 |
|  |  |  | **Sham** | 22.31  (13.25) | 27.82  (14.07) | 28.05  (15.18) |  | - | - | - |  |  |  |  |  |
|  |  | **High** | **Anodal** | 16.98  (7.45) | 28.70  (23.02) | 30.83  (25.74) | 0.007 | - | - | - | 0.972 | - | - | - | 0.913 |
|  |  |  | **Sham** | 15.02  (8.70) | 27.77  (19.80) | 32.84  (24.97) |  | - | - | - |  |  |  |  |  |
|  | **Beta** | **Low** | **Anodal** | 6.83  (3.62;13.20) | 8.46  (5.11;14.20) | 8.69  (7.29;15.77) | **0.030** | 0.137  (0.36) | 0.037  (0.53) | 1.000  (0.20) | - | 0.950  (0.08) | 0.950  (0.07) | 0.950  (0.07) | - |
|  |  |  | **Sham** | 7.73  (4.95;11.12) | 9.32  (5.45;13.49) | 9.29  (5.61;16.96) | 0.142 | - | - | - |  |  |  |  |  |
|  |  | **High** | **Anodal** | 6.44  (2.34) | 8.17  (4.24) | 9.85  (5.84)) | 0.003 | - | - | - | 0.965 | - | - | - | 0.526 |
|  |  |  | **Sham** | 5.58  (2.93) | 9.59  (6.09) | 9.59  (5.60) |  | - | - | - |  |  |  |  |  |

*Statistically significant after Bonferroni’s correction (*p* < 0.016), Bolded number indicate statistically significant.

**Additional data 5** Means raw scores of motor outcomes (FMA-UE, FMA-LE, WMFT-pencil, and WMFT-can) and statistical analysis.

| **Outcomes** | **Analysis of** | **Groups** | **Means (SD) ^a^** | | | **Within group comparison ^b^**  ***p*-value (effect size)** | | | | **Between groups comparison ^b^**  ***p*-value (effect size)** | | | **Interaction effect (Time x Group)** |
| --- | --- | --- | --- | --- | --- | --- | --- | --- | --- | --- | --- | --- | --- |
|  |  |  | **Pre** | **Post** | **F/U** | **Overall** | **Pre vs Post** | **Pre vs F/U** | **Post vs F/U** | **Overall** | **At Post** | **At F/U** |  |
| FMA-UE | All | Anodal | 36.47 (26.91) | 44.60 (25.32) | 49.53 (25.45) | **<0.001** | 0.019  (1.60) | **<0.001***  **(1.17)** | 0.513  (0.42) | - | 0.453  (0.25) | 0.787  (0.24) | - |
|  |  | Sham | 38.47 (24.99) | 44.87 (25.83) | 48.53 (23.83) | **<0.001** | **0.002***  (1.38) | **<0.001***  **(1.38)** | 1.000  (0.42) |  |  |  |  |
|  | Low impairment | Anodal | 60.13 (2.64) | 65.50 (0.53) | 66.00 (0.00) | <0.001 | - | - | - | 0.136 | - | - | 0.099 |
|  |  | Sham | 61.67 (2.80) | 65.00 (1.26) | 64.67 (1.51) |  | - | - | - |  |  |  |  |
|  | High impairment | Anodal | 9.43 (9.09) | 20.71 (15.72) | 30.71 (27.13) | 0.085 | - | - | - | - | 0.669  (0.34) | 0.634  (0.44) | - |
|  |  | Sham | 23.00 (20.38) | 31.44 (25.68) | 37.78 (25.82) | **0.004** | 0.102  (1.57) | **0.004***  **(1.92)** | 0.867  (0.68) |  |  |  |  |
| FMA-LE | All | Anodal | 22.93 (12.00) | 25.87 (10.87) | 28.33 (8.28) | **<0.001** | 0.067  (1.32) | **<0.001***  **(1.45)** | 0.249  (0.64) | - | 0.083  (0.55) | 0.201  (0.65) | - |
|  |  | Sham | 24.00 (11.21) | 25.33 (11.53) | 26.47 (10.53) | **0.004** | 0.134  (1.00) | **0.010***  **(1.52)** | 1.000  (0.62) |  |  |  |  |
|  | Low impairment | Anodal | 31.13 (2.17) | 33.00 (0.93) | 33.63 (0.52) | **0.004** | **0.005***  **(1.22)** | **<0.001***  **(1.65)** | 0.745  (0.29) | 0.080 | 0.087  (0.81) | 0.010  (0.89) | **0.040** |
|  |  | Sham | 33.00 (0.89) | 33.50 (0.84) | 33.33 (0.82) |  | 1.000  (1.29) | 1.000  (2.24) | 1.000  (0.85) |  |  |  |  |
|  | High impairment | Anodal | 13.57 (11.77) | 17.71 (11.37) | 22.29 (8.92) | **0.021** | 0.247  (1.54) | 0.023  (2.23) | 1.000  (1.10) | - | 0.120  (0.65) | 0.126  (1.19) | - |
|  |  | Sham | 18.00 (10.87) | 19.89 (12.20) | 21.89 (11.61) | **0.019** | 0.377  (1.21) | 0.020  (2.10) | 0.716  (0.82) |  |  |  |  |
| WMFT-Pencil | All | Anodal | 57.78 (60.25) | 49.05 (59.97) | 41.13 (57.74) | **<0.001** | 0.301  (0.41) | **0.003***  **(0.58)** | 0.301  (0.22) | - | 0.592  (0.02) | 0.220  (0.24) |  |
|  |  | Sham | 49.45 (59.63) | 41.35 (57.57) | 41.26 (57.64) | **0.008** | 0.082  (0.39) | 0.085  (0.39) | 1.000  (0.00) |  |  |  |  |
|  | Low impairment | Anodal | 3.31(1.60) | 1.66(0.44) | 1.35(0.40) | <0.001 | - | - | - | 0.101 | - | - | 0.085 |
|  |  | Sham | 2.37(0.99) | 1.66(0.70) | 1.57(0.53) |  | - | - | - |  |  |  |  |
|  | High impairment | Anodal | 120.00 (0.00) | 103.20 (44.45) | 86.59 (57.07) | 0.620 | - | - | - | - | 0.898  (0.09) | 0.761  (0.43) | - |
|  |  | Sham | 80.84 (58.74) | 67.82 (61.89) | 67.71 (62.01) | 0.814 | - | - | - |  |  |  |  |
| WMFT-can | All | Anodal | 57.34 (60.67) | 49.19 (59.85) | 33.19 (54.19) | **<0.001** | 0.134  (0.39) | **0.002***  **(0.71)** | 0.432  (0.40) | - | 0.799  (0.01) | 0.602  (0.01) |  |
|  |  | Sham | 49.67 (59.45) | 41.36 (57.56) | 26.06 (48.64) | **0.004** | 0.432  (0.40) | **0.010***  **(0.71)** | 0.432  (0.39) |  |  |  |  |
|  | Low impairment | Anodal | 2.52(1.16) | 1.54(0.41) | 1.31(0.31) | **<0.001** | 0.037  (1.52) | **0.001***  **(1.67)** | 0.952  (0.24) | - | 0.414  (0.39) | 0.059  (0.64) | - |
|  |  | Sham | 2.23(0.78) | 1.77(0.55) | 1.75(0.49) | 0.252 | - | - | - |  |  |  |  |
|  | High impairment | Anodal | 120.00 (0.00) | 103.65 (43.27) | 69.62 (62.83) | 0.006 | - | - | - | 0.751 | - | - | 0.903 |
|  |  | Sham | 81.29 (58.06) | 67.76 (61.95) | 42.27 (58.32) |  | - | - | - |  |  |  |  |

For statistical analysis, absolute change scores (∆) from individual PRE data were used for analysis, and the calculated formulas were as follows: 1) baseline = |PRE-PRE|, 2) at POST = |POST-PRE|, and 3) at F/U = |F/U-PRE|. ^a^ raw scores data (Full score of FMA-UE =66 and FMA-LE = 33), ^b^ statistical analysis from change scores, Cohen’s values of 0.20, 0.50, and 0.80 were interpreted as small, moderate, and large effect sizes, respectively. *Statistically significant after Bonferroni’s correction (*p* < 0.016), Bolded number indicate statistically significant.

**Supplement data 6**

Types of training after intervention

Summary of received rehabilitation details after intervention were shown in **Table A**. Fisher exact test reported no significant difference between the groups.

**Table A** Summary of received rehabilitation after intervention

| **Variable** | **Anodal group**  **(n=15)** | **Sham group (n=15)** | ***p*-value** |
| --- | --- | --- | --- |
| Rehabilitation (PT/self-home exercise) | 4/11 | 5/10 | 1.000 |
| PT program |  |  | 0.524 |
| - Thrice a week | 2 | 1 |  |
| - 7 days/week   (Intensive program) | 2 | 4 |  |
